# Prognostic Roles of Cross-Talk between Peritumoral Hepatocytes and Stromal Cells in Hepatocellular Carcinoma Involving Peritumoral VEGF-C, VEGFR-1 and VEGFR-3

**DOI:** 10.1371/journal.pone.0064598

**Published:** 2013-05-30

**Authors:** Peng-Yuan Zhuang, Jun Shen, Xiao-Dong Zhu, Lu Lu, Lu Wang, Zhao-You Tang, Hui-Chuan Sun

**Affiliations:** 1 Department of General Surgery, Xinhua Hospital, School of Medicine, Shanghai Jiao Tong University, Shanghai, People’s Republic of China; 2 Liver Cancer Institute and Zhongshan Hospital, Fudan University, Shanghai, People’s Republic of China; Virginia Commonwealth University, United States of America

## Abstract

**Background:**

Peritumoral liver tissue could play a potential role in hepatocellular carcinoma (HCC) progression and patient survival via angiogenesis- and lymphangiogensis-related factors. The prognostic role of these factors in hepatocytes and stromal cells in HCC patients after curative resection remains to be explored.

**Methods:**

Tumor tissue and surrounding peritumoral tissue were obtained from 145 resected HCC patients without lymph node metastasis (LNM) and 37 resected HCC patients with LNM. Tissue microarrays were constructed from duplicate cores of tumor tissue and surrounding peritumoral tissue from each resected specimen. Immunohistochemistry and real-time polymerase chain reaction were used to evaluate the expression of vascular endothelial growth factor-A (VEGF-A), VEGF-C, VEGF receptor-1(VEGFR-1), VEGFR-2, and VEGFR-3. Macrophage infiltration was determined by CD68 staining. Correlations between the expression of these factors and overall survival (OS) and time to recurrence (TTR) were studied.

**Results:**

The peritumoral expression of VEGF-A, VEGF-C, VEGFR-1, VEGFR-2, and VEGFR-3 were significantly higher than expression of these factors in tumors. VEGFR-1 was mostly located in peritumoral macrophages, while VEGF-C and VEGFR-3 were mostly located in peritumoral hepatocytes. HCC with high peritumoral co-expression of VEGF-C, VEGFR-1, and VEGFR-3 was associated with higher peritumoral distribution of macrophages (0.87%±0.26% versus 0.45%±0.20%), LNM (32.4% versus 12.0%), shorter TTR (10.2 months versus 34.5 months), and poor prognosis (19.4 months versus 49.3 months).

**Conclusion:**

Expression of VEGF-C, VEGFR-1, and VEGFR-3 in peritumoral liver tissue is associated with a unique type of HCC that has a poorer outcome after hepatectomy.

## Introduction

The vast majority of hepatocellular carcinoma (HCC) deaths are caused by the formation of metastases [1], [2], [3]. As in other tumor types, the selective process of metastasis in HCC requires active cross-talk between tumor cells and peritumoral tissue, which is mediated by direct tumor cell–stromal cell contact or paracrine cytokine and growth factor signaling such as peritumoral macrophage colony-stimulating factor (M-CSF) and macrophages as reported in our previous study [4]. This cross-talk is associated with HCC progression, tumor recurrence, and patient survival after hepatectomy [4], [5]. Additionally, Budhu et al. [6]showed that intrahepatic venous metastasis was associated with a unique immune or inflammatory response signature in peritumoral liver tissue. Therefore, the peritumoral environment should be fully taken into account in assessing the process of HCC progression.

Angiogenesis and lymphangiogenesis exert important roles in HCC growth and metastasis. In particular, vascular endothelial growth factor-A (VEGF-A) and its receptors such as VEGF receptor-1(VEGFR-1) and VEGFR-2 have been implicated in induction of tumor-associated angiogenesis in HCC, while the VEGF-C/VEGFR-3 axis has been shown to be involved in lymphangiogenesis and subsequent lymph node metastasis (LNM) [7], [8], [9], [10], [11], [12]. However, most research has focused on the intratumoral environment, and the potential roles of angiogenesis and lymphangiogensis in the peritumoral environment remain unclear. Furthermore, the stromal cells in peritumoral liver tissue, including VEGFR-1–expressing macrophages, monocytes, and fibroblasts, could also play a role in HCC progression [13], [14].

In the present study, using tissue microarrays (TMAs) and distant peritumoral liver tissue, as well as immunohistochemistry staining and real-time polymerase chain reaction (PCR), we demonstrated interaction between peritumoral hepatocytes and stromal cells, particularly macrophages, that may affect HCC recurrence and patient survival.

## Materials and Methods

### Patients and Treatment Protocol

From January 1999 through March 2006, 968 patients underwent curative resection for HCC. Information on these patients formed a prospectively collected database, from which we randomly selected 182 subjects (**Table 1**). One hundred forty-five patients (132 men, 13 women, median age 51 years, range 25–75 years) did not have LNM, while 37 patients (34 men, 3 women, median age 51 years, range 27–71 years) had pathology-proven HCC and LNM; 20 of these latter patients underwent lymphadenectomy. None of the patients received any preoperative anticancer treatment. The preoperative liver function in all patients was classified as Child A stage. Tumor stage was determined according to the UICC TNM classification system (7th edition). Tumor differentiation was graded by the Edmondson grading system. The Scheuer system was applied for grading (necroinflammatory activity in chronic hepatitis) and staging (fibrosis and cirrhosis) of the peritumoral liver tissue [15], [16].

**Table 1:** Clinicopathologic features of patients from three cohorts.

Feature	Cohort 1(182 patients)	Cohort 2(45 patients)	Cohort 3(35 patients)
Age (years), median (range)	52 (16–75)	51 (23–72)	51(31–68)
Sex, female/male	16/166	5/40	4/31
Preoperative ALT, U/L, mean ± SD	46.8±32.3	42.0±31.8	44.3±30.2
α-Fetoprotein, ng/mL, mean ± SD	4535.3±2340.7	4687.4±2102.3	4320.3±1973.4
Liver cirrhosis, no/yes	45/137	7/38	5/30
HBsAg, no/yes	38/144	9/36	6/29
Tumor size, cm, mean ± SD	6.6±5.0	6.4±4.6	6.1±4.8
Lymph node metastasis, no/yes	145/37	42/3	33/2
Satellite lesions, no/yes	146/36	40/5	30/5
Cancerous thrombi, no/yes	111/71	31/14	23/12
Tumor differentiation, I–II/III–IV	135/47	39/6	32/3
TNM stage, I/II/IIIA	117/23/42	32/6/7	31/2/2

Abbreviations: ALT, alanine aminotransferase; HBsAg, hepatitis B surface antigen;

Our approach for hepatectomy in HCC cases has been described previously [17], [18]. Briefly, the indications for hepatectomy are that the main tumor is technically resectable, no cancerous thrombi are present in the main trunk of the portal vein, and no distant metastasis to other organs has occurred. Lymphadenectomy is conducted according to the intraoperative resectability of enlarged lymph nodes (LNs), whereas incisional or aspiration biopsy of LNs in certain strictly defined circumstances is performed to obtain a postoperative histologic diagnosis and to avoid any further operative risks. LN detection had no impact on the resection type among our subjects. For postoperative treatment of LNM, radiotherapy or chemotherapy is given to patients with nonresectable metastatic LNs based on surgeon preference and patient consent. Patients who undergo lymphadenectomy receive no special treatment until recurrence is diagnosed. In our study, recurrence included intrahepatic liver recurrence and metastasis to the lung and other organ. We confirm that the research has been conducted in compliance with the appropriate ethical guidelines of the declaration of Helsinki. The study was approved by the local ethics committee at the faculty of medicine, University of Jiao Tong. All subjects were written informed about the background of the study and anonymity of data collection. We confirm that we obtained informed written consent from all participants involved in the study.

### Follow-up

Patients were followed up in our clinic every 2 months during the first postoperative year and at least every 3–4 months afterward. Liver function, α-fetoprotein, and hematologic parameters were examined, and liver ultrasonography was done by independent doctors who had no knowledge of the study. A computed tomography (CT) scan of the chest and abdomen was performed every 6 months. Bone scanning or magnetic resonance imaging (MRI) was done if localized bone pain was reported. If recurrence was suspected, CT scanning or MRI was performed immediately. The median follow-up time was 21.3 months.

### Tissue Microarray and Immunohistochemistry

After reviewing hematoxylin and eosin (HE)-stained slides to locate the tumor tissue and tissue adjacent to tumor (TAT) within 2 cm from tumor, we constructed TMA slides in collaboration with Shanghai Biochip Company, Ltd. (Shanghai, China). Two cores were taken from each formalin-fixed, paraffin-embedded HCC and TAT sample, respectively, by using punch cores that measured 1.0 mm in diameter from the nonnecrotic area of tumor foci and TAT. Immunohistochemistry was performed by a two-step method using a primary antibody and heat-induced antigen-retrieval procedures. Sections were incubated overnight at 4°C with primary antibody. After excess primary antibody was washed off, the components of the Envision-plus detection system were applied with an anti-mouse polymer (EnVision+/HRP/Mo, Dako, Glostrup, Denmark). Reaction products were visualized by incubation with 3,3′-diaminobenzidine. The following primary antibodies were used: mouse monoclonal VEGF-A (1∶100, DAKO) rabbit polyclonal VEGF-C (1∶100, Santa Cruz Biotechnology, Santa Cruz, CA), mouse monoclonal VEGFR-1 (1∶50, Santa Cruz Biotechnology), mouse monoclonal VEGFR-2 (1∶100, Santa Cruz Biotechnology), rabbit polyclonal VEGFR-3 (1∶20, Santa Cruz Biotechnology), mouse monoclonal CD68 for staining of macrophages (1∶100, Zymed Laboratories, San Francisco, CA), rabbit polyclonal CD31 for staining of blood vessels (1∶200, Abcam, Cambridge, MA), and mouse monoclonal D2-40 for staining of lymphatic vessels (1∶200, Abcam). Negative controls were treated identically except omission of the primary antibody.

### Real-Time Polymerase Chain Reaction

Expression of VEGF-C mRNA, VEGF-A mRNA, VEGFR-1 mRNA, VEGFR-2 mRNA, and VEGFR-3 mRNA in another independent cohort of 45 paired tumors and peritumoral tissues was evaluated by real-time reverse-transcription PCR (**Table 1**). Total RNA was extracted with TRIzol (Invitrogen, Carlsbad, CA), and 1 µg total RNA was reverse-transcribed by using the Primescript RT reagent kit (Takara Bio, Tokyo, Japan). Real-time PCR for quantification was performed using SYBR Premix Ex Taq (Takara Bio). The reactions were performed in triplicate. The expression level of all five factors was normalized to the expression level of β-actin, a housekeeping gene control. Primer sequences were as follows: forward primer 5′- ATTTGCTGCAGCACATTATAATACAGAGAT -3′ and reverse primer 5′-TCACTATATGAAAATCCTGGCTCACAAGCC-3′ for human VEGF-C; forward primer 5′-GCAAGACAAGAAAATCCCTG-3′ and reverse primer 5′-GGCTTGTCACATCTGCAA-3′ for human VEGF-A; forward primer 5′-TCACTGCCACTCTAATTGTC-3′ and reverse primer 5′- CCATATGCGGTACAAGTCA-3′ for human VEGFR-1; forward primer 5′- AAGGCGAGACCTGCATTC-3′ and reverse primer 5′- CTGCCCTCTTCTGAGCTCT-3′ for human VEGFR-2; forward primer 5′- AGCCATTCATCAACAAGCCT-3′ and reverse primer 5′- GGCAACAGCTGGATGTCATA-3′ for human VEGFR-3; forward primer 5′- CATCTCTTGCTCGAAGTCCA-3′ and reverse primer 5′- ATCATGTTTGAGACCTTCAACA-3′ for human β-actin. The relative amount of tissue mRNA, standardized by the amount of β-actin mRNA, was expressed as −ΔCT = [CT (factor) − CT (β-actin)]. The ratio of the number of mRNA copies to the number of β-actin mRNA copies was then calculated as 2 − ΔCT×K, where K is a constant.

### Immunofluorescent Staining

Primary antibodies for immunofluorescent staining were a mouse monoclonal CD68 (1∶100, Zymed Laboratories, San Francisco, CA), a rabbit monoclonal VEGFR-1 antibody (1∶250, Abcam). Primary antibodies were detected by using secondary antibodies of anti-mouse IgG-TR (Santa Cruz Biotechnology) and anti-rabbit IgG-FITC (Santa Cruz Biotechnology), respectively. Frozen peritumoral liver sections (8 µm) were air-dried, hydrated with PBS, blocked with 10% goat serum in PBS for 30 min, and incubated with primary antibodies overnight at 4°C. Sections were washed three times in PBS, followed by secondary antibody for 1 h at room temperature. After washing in PBS, sections were mounted with anti-fade reagent with 4′,6-diamidino-2-phenylindole (DAPI) (Invitrogen) and viewed with fluorescent microscope (×20 objective magnification, Olympus).

### Evaluation of Immunohistochemical Findings

The density of positive staining was measured using a computerized image system composed of a Leica CCD camera DFC420 (Leica Microsystems Imaging Solutions, Ltd., Cambridge, UK), connected to a Leica DM IRE2 microscope (Leica Microsystems Imaging Solutions, Ltd.). Under high-power view, the pictures of four representative fields were captured by the Leica QWin Plus v3 software (Leica Microsystems Imaging Solutions) at a setting identical to the image system. All the biomarkers were counted by Image-Pro Plus v6.2 software (Media Cybernetics, Inc., Bethesda, MD). For examining the staining for each antibody, we used the same setting for all slides. Integrated optical density (IOD) in each picture was measured. For the quantification of mean vessel density in sections stained for CD31, five fields at ×100 magnification in the “hotspot” were captured for each tumor and microvessel density (MVD) was quantified as CD31-positive area/total area, and lymphatic vessel density (LVD) was also quantified as the D2-40-positive area/total area; CD68-positive areas were measured by Leica Qwin Plus on the pictures, and macrophage density was formulated as CD68-positive area/total area of each picture (400×).

### Distant Peritumoral Sections

Another cohort of 35 independent specimens of distant peritumoral tissue (at least 30 mm from the tumor edge) from patients that underwent hepatectomy for HCC (**Table 1**) were collected and then immunostained with VEGF-C, VEGFR-1, and VEGFR-3 antibodies. Peritumoral tissues at three distances (5, 15, and 25 mm) away from the tumor margin were observed, and pictures of three hotspots (200×) at each distance were taken by the computerized imaging system already described. Measurement of expression of VEGF-C, VEGFR-1, and VEGFR-3 was performed as described.

### Data Analysis

OS or time to recurrence (TTR) was defined as the interval between surgery and death or recurrence. For 17 patients with LNM who didn’t receive lymphadenectomy, the recurrence lesions were diagnosed with the exception of the residual metastatic LNs.

Analysis was performed with SPSS 15.0 for Windows (SPSS, Chicago, IL); Spearman rank correlation coefficient determination was used to analyze the correlation among parameters. Univariate analysis of variance was used to analyze the distributions of biomarkers in long-distance peritumoral liver tissue. Kaplan-Meier analysis and Log-rank test was used to compare OS and TTR, and *p*<0.05 was considered statistically significant.

## Results

### Immunohistochemistry of VEGF-A, VEGF-C, VEGFR-1, VEGFR-2, and VEGFR-3 in HCC and Peritumoral Tissues

**Figure 1:**
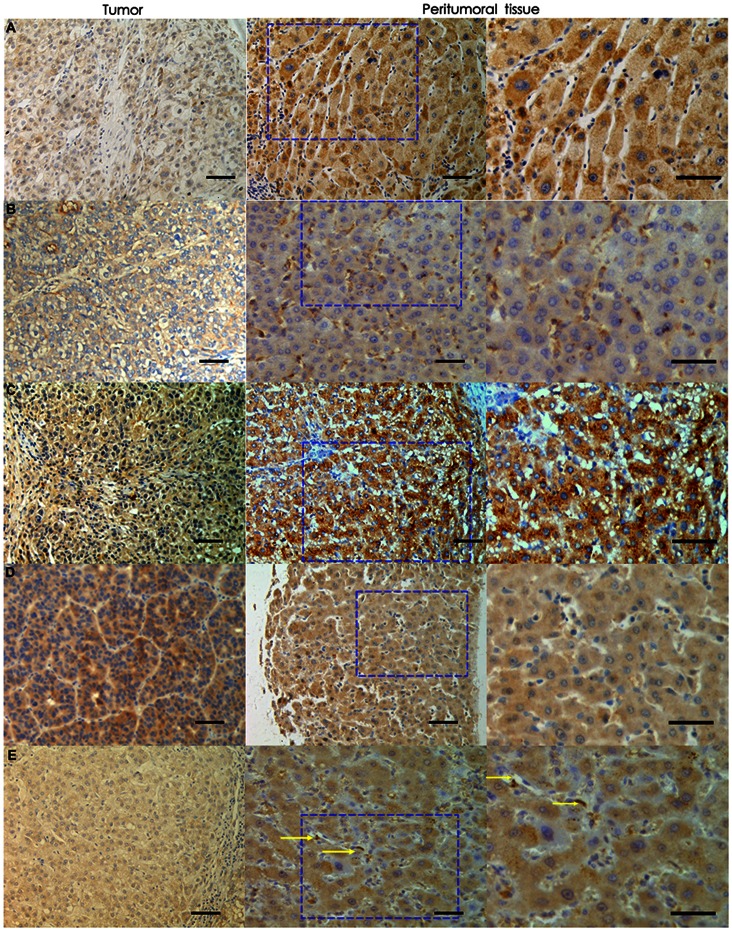
Peritumoral and intratumoral expression of VEGF-A, VEGF-C, VEGFR-1, VEGFR-2, and VEGFR-3. Peritumoral expression of VEGF-A, VEGF-C, VEGFR-1, VEGFR-2, and VEGFR-3 was much higher than expression of these factors in tumor tissue. (*A:* VEGF-A; *B:* VEGFR-1; *C:* VEGFR-2; *D:* VEGF-C; *E:* VEGFR-3; 200×; stromal cells are indicated by arrows in *E*).Black bars, 50um).

We evaluated the expression of VEGF-C, VEGF-A, VEGFR-1 VEGFR-2, and VEGFR-3 in HCC and peritumoral tissues in 182 HCC patients, and observed a higher expression level of all five factors in peritumoral tissue than in tumor tissue (**Table 2**). This finding was further validated by the mRNA expression levels in the independent cohort of 45 HCC patients (**Table 2**). In peritumoral tissue, VEGF-A, VEGF-C, and VEGFR-2 were detected in the hepatocytes, VEGFR-3 was detected mostly in the hepatocytes and weakly in stromal cell, while most VEGFR-1 was expressed in stromal components. (**Figure 1**).

**Table 2:** Expression of the five factors in tumors and peritumoral tissue in HCC.

Protein level (cohort 1, *n* = 182)			
	Tumor tissue	Peritumoral tissue	*p*
VEGF-A	61352.4±6574.1^a^	116769.2±66781.5^a^	0.000
VEGFR-1	563672.3±212032.5	787402.3±211323.4	0.002
VEGFR-2	23234.7±7342.2	586098.5±21211.2	0.000
VEGF-C	2554342.4±143453.2	4281456.5±234654.2	0.004
VEGFR-3	1526352.6±252564.2	2240063.4±343563.5	0.003
**mRNA level (independent cohort 2, ** ***n*** ** = 45)**			
VEGF-A	−7.3±0.13^b^	−4.5±0.23^b^	0.003
VEGFR-1	−4.1±0.09	−2.7±0.11	0.001
VEGFR-2	−11.3±0.23	−3.5±0.13	0.000
VEGF-C	−9.2±0.17	−6.0±0.22	0.005
VEGFR-3	−10.3±0.26	−8.5±0.17	0.000

a Values were measured as integrated optical density (IOD).

b Values were expressed as –△CT = [CT (factor) –CT (β-actin)].

Abbreviations: VEGF, vascular endothelial growth factor; VEGFR, VEGF receptor.

To explore the role of the five factors in peritumoral tissue, we classified patients into two groups using median value (IOD) as a cutoff. The median values for each factor were as follows: 102,557.2 for VEGF-A, 688,324.2 for VEGFR-1, 578,863.1 for VEGFR-2, 4,279,934.3 for VEGF-C, and 2,238,002.5 for VEGFR-3.

### High Peritumoral Co-expression of VEGF-C, VEGFR-1, and VEGFR-3 Was Correlated with Poor Prognosis and Early Recurrence

**Figure 2:**
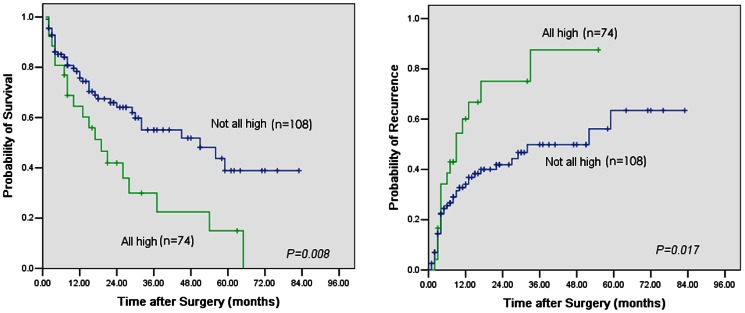
High peritumoral co-expression of VEGF-C, VEGFR-1, and VEGFR-3 was correlated with poor prognosis and early recurrence. **Left:** Cumulative overall survival (OS) curves show that patients with high peritumoral co-expression of VEGF-C, VEGFR-1, and VEGFR-3 had poorer OS (Kaplan-Meier, log-rank, *p* = 0.008). **Right:** Time to recurrence (TTR) curves showed that patients with high peritumoral co-expression of VEGF-C, VEGFR-1, and VEGFR-3 had shorter TTR (Kaplan-Meier, log-rank, *p* = 0.017).

Among the 182 patients up to the last follow-up, 89 patients had tumor recurrence and 53 patients died, including 15 patients that died of liver failure without tumor recurrence. We studied different combination of the five factors based on the stromal expression (VEGFR-1, VEGFR-3) and the hepatocyte expression (VEGF-A, VEGFR-2, VEGF-C, VEGFR-3). We found that patients with high peritumoral co-expression of VEGF-C, VEGFR-1, and VEGFR-3 had the poorest prognosis compared with patients with different expression combinations (**Table S1**). The results showed that 74 patients (40.7%) with high peritumoral co-expression of VEGF-C, VEGFR-1, and VEGFR-3 had both poorer OS and shorter TTR compared with patients that had different expression patterns of VEGF-C, VEGFR-1, and VEGFR-3 (median OS: 19.4 months versus 49.3 months, *p* = 0.008; median TTR: 10.2 months versus 34.5 months, *p* = 0.017) (**Figure 2**). Moreover, patients with high peritumoral expression of one or two of the factors VEGF-C, VEGFR-1, and VEGFR-3 had a prognosis similar to those with low peritumoral expression of all three factors (**Table S2**). By dividing all recurrent cases into early or late recurrence groups, using 1 year as the cutoff value of TTR as suggested by Poon et al. [19], [20], we found that patients with high peritumoral co-expression of VEGF-C, VEGFR-1, and VEGFR-3 had a higher incidence of early recurrence (56.8%, 42/74 versus 25.9%, 28/108, *p* = 0.000) than later recurrence (10.8%, 8/74, versus 10.2%, 11/108, *p* = 0.892); furthermore, using 2 year after resection as the discriminant of the prognosis of early and later recurrence, we also found that patients with high peritumoral co-expression of VEGF-C, VEGFR-1, and VEGFR-3 also had a higher incidence of early recurrence (59.4%, 44/74 versus 26.9%, 29/108, p = 0.000) than later recurrence (8.1%, 6/74, versus 9.3%, 10/108, p = 0.788).

Univariate and multivariate analyses were performed to determine the risk factors of OS and TTR (**Table 3** **,** **4**). In univariate analyses, presence of LNM, satellite lesions, cancerous thrombi, liver cirrhosis, larger tumor size, and presence of high peritumoral co-expression of the three factors was associated with OS, and all these factors except for cirrhosis were associated with TTR. In the multivariate analyses, we found that high co-expression of the three markers in peritumoral liver tissues was an independent factor for OS (HR: 2.018; 95% CI: 1.124–2.804; *p* = 0.028) and TTR (HR: 1.570; 95% CI: 1.130–1.942; *p* = 0.022).

**Table 3:** Univariate analyses of factors associated with survival and recurrence.

	OS (*p*)	TTR (*p*)
Lymph node metastasis: no vs. yes	<0.001	0.005
Satellite lesions: no vs. yes	<0.001	<0.001
Tumor size (cm): ≤5 vs. >5	<0.001	<0.001
Cancerous thrombi: no vs. yes	<0.001	<0.001
Cirrhosis nodules: no vs.yes	0.013	0.057
High peritumoral co-expression of three factors: no vs. yes	0.008	0.017

Abbreviations: OS, overall survival; TTR, time to recurrence.

**Table 4:** Multivariate analyses of factors associated with survival and recurrence.

	HR	95% CI	*p*
Overall survival			
Lymph node metastasis	1.532	0.986–2.401	0.075
Satellite lesions	1.452	1.230–1.860	0.007
Tumor size	2.830	1.563–4.580	<0.001
Cancerous thrombi	1.848	1.429–2.543	<0.001
Cirrhosis nodules	2.791	0.940–3.540	0.042
High peritumoral co-expression of three factors	2.018	1.124–2.804	0.028
Time to recurrence			
Lymph node metastasis	2.140	1.280–3.520	0.012
Satellite lesions	1.567	1.238–2.010	<0.001
Tumor size	1.329	1.122–1.598	0.008
Cancerous thrombi	1.411	1.130–1.745	0.003
High peritumoral co-expression of three factors	1.570	1.130–1.942	0.022

Abbreviations: HR, hazard ratio; CI, confidence interval.

### VEGF-C, VEGFR-1, and VEGFR-3 Distribution in Peritumoral Liver Tissue

**Figure 3:**
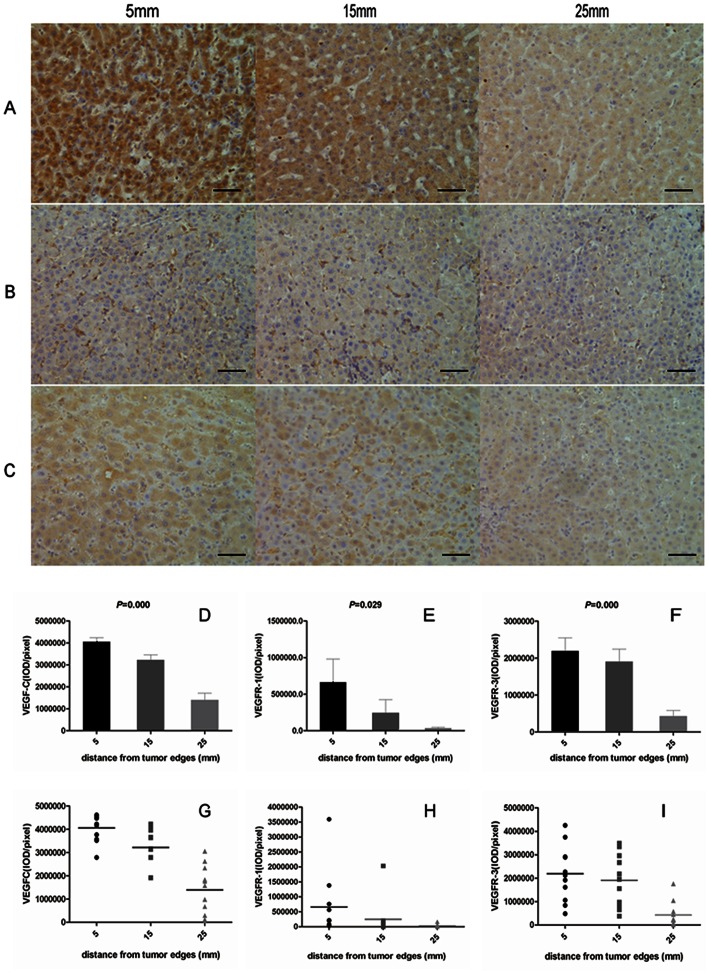
VEGF-C, VEGFR-1, and VEGFR-3 Distribution in Peritumoral Liver Tissue. Representative distant peritumoral liver sections of VEGF-C (*A*), VEGFR-1 (*B*), and VEGFR-3 (*C*) staining. All three factors decreased with distance from the tumor edge. D–F showed an obviously down-regulation of all three factors along the tumor marginal distances for VEGF-C (*D*), VEGFR-1 (*E*), and VEGFR-3 (*F*), respectively. Peritumoral expression of the three factors differed in 35 patients for VEGF-C (*G*), VEGFR-1 (*H*), and VEGFR-3 (*I*). Black bars, 50 um.

Immunohistochemistry of the distant section (**Figure 3**) revealed a graded distribution of VEGF-C, VEGFR-1, and VEGFR-3 peritumoral expression, with the expression of each factor decreasing as the distance from the tumor margin increased. At 5, 15, and 25 mm, the average expression intensity (IOD) was 4,058,037.7±175,491.5, 3,216,064.4±245,064.2, and 1,392,977.2±313,326.6 for VEGF-C; 660,825.2±317,651.1, 243,649.4±180,269.3, and 30,737.2±14,862.0 for VEGFR-1; 2,189,577.0±358,548.8, 1,904,552.4±337,152.3, and 421,964.0±163,019.1 for VEGFR-3 (**Figure 3**), respectively. Univariate analysis showed decreased expression of all three factors when the distance from the tumor margin increased (*p* = 0.000, *p* = 0.029, and *p* = 0.000 for VEGF-C, VEGFR-1, and VEGFR-3, respectively), which was also associated with the distance (Spearman’s correlation test, correlation coefficient *(cc)* = 0.807, *p* = 0.000 for VEGF-C; *cc* = 0.647, *p* = 0.000 for VEGFR-1; *cc* = 0.643, *p* = 0.000 for VEGFR-3).

### Location of Peritumoral Expression of VEGF-C, VEGFR-1, and VEGFR-3

**Figure 4:**
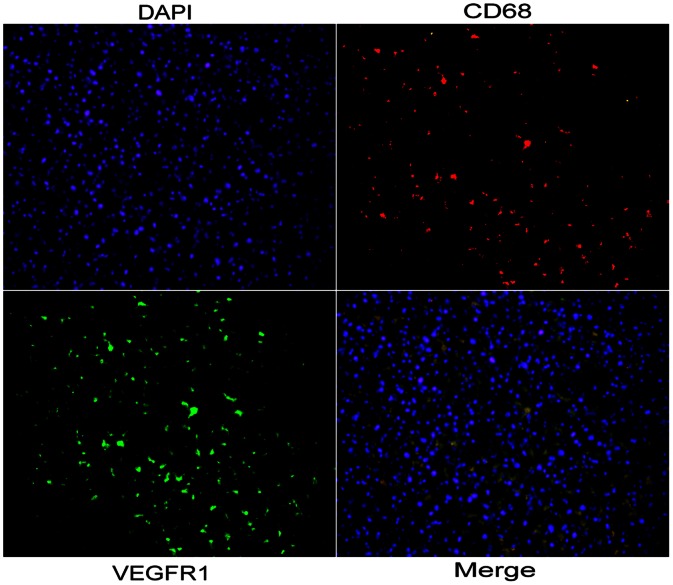
Location of peritumoral expression of VEGFR-1. Co-expression of CD68 and VEGFR-1 in stromal compartments in peritumoral liver tissue. The CD68 (red) and VEGFR-1 (green) signals were due to rhodamine- and FITC-labeled antibodies, respectively, using single-layer projections in a confocal microscope. Hepatocyte nuclei were labeled by DAPI (blue).

In peritumoral tissue, the expression of VEGF-C and VEGFR-3 were significantly correlated (*cc* = 0.348, *p* = 0.000), both were mainly located in peritumoral hepatocytes, and high peritumoral expression of VEGF-C, VEGFR-1, and VEGFR-3 was associated with higher peritumoral macrophages density as determined by CD68 staining (0.87%±0.26% versus 0.45%±0.20%, *p* = 0.000, as compared with the rest of the patients). Moreover, based on double immunofluorescence methods, we found that most of the CD68-positive macrophages were VEGFR-1 positive (**Figure 4**).

### Clinicopathological Features of High Peritumoral Co-expression of VEGF-C, VEGFR-1, and VEGFR-3

Of the 182 patients with HCC, the 74 patients with high peritumoral expression of the three factors tended to have larger tumor size (10.2±3.7 cm versus 6.2±3.5 cm, *p* = 0.006 ); however, no correlation was observed in terms of age, sex, TNM staging system, or other tumor characteristics. Furthermore, we did not find expression of the three factors to be associated with inflammatory status of peritumoral liver tissue, including necroinflammatory activity (grade) and cirrhosis (stage) scores as well as hepatitis B surface antigen and preoperative serum alanine aminotransferase level (**Table 5**).

**Table 5:** The clinicopathologic factors related to HCC with high peritumoral co-expression of VEGF-C, VEGFR-1, and VEGFR-3.

HCC (*n* = 182)			
	Co-expression (−)*n* = 108 (%)^a^	Co-expression (+)*n* = 74 (%)^a^	*p*
Age (years)^b^	52.8±10.1	49.0±11.5	0.187
Sex, female/male	10/98 (90.7)	6/68 (91.9)	0.788
HBsAg, no/yes	23/85 (78.7)	15/59 (79.7)	0.867
Preoperative ALT (U/L)^b^	47.8±32.7	45.3±48.2	0.864
α-Fetoprotein (ng/mL)^b^	4343.3±2132.7	4654.9±2760.7	0.740
Cirrhosis, no/yes	28/80 (74.1)	17/57 (75.7)	0.650
Tumor size (cm)^b^	6.2±3.5	10.2±3.7	0.006
Satellite lesion, no/yes	86/22 (20.4)	60/14 (18.9)	0.809
Vascular invasion, no/yes	66/42 (38.9)	45/29 (39.2)	0.967
Lymph node metastasis, no/yes	95/13	50/24	0.001
TNM stage, I/II/IIIA	69/16/23 (21.3))	48/7/19 (25.7)	0.505
Edmondson grade, I–II/III–IV	81/27 (25.0)	54/20 (27.0)	0.759
Scheuer’s score			
Grade, 1–2/3–4	63/45 (41.7)	41/33 (44.6)	0.695
Stage, 1–3/4	65/43 (39.8)	44/30 (40.5)	0.922
Peritumoral macrophage density^b^	0.45%±0.20%	0.87%±0.26%	0.000
Microvessel density^b^	7.72%±0.45%	7.78%±0.40%	0.870
Lymphatic vessel density^b^	0.32%±0.25%	0.66%±0.20%	0.012

a The proportion of the latter clinicopathologic parameter.

b Student *t* test.

Abbreviations: ALT, alanine aminotransferase; HBsAg, hepatitis B surface antigen;

**Figure 5:**
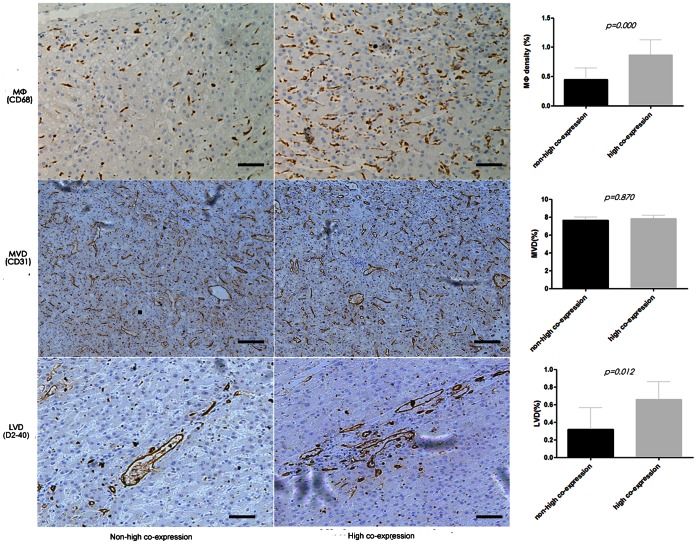
Macrophages infiltration, LVD and MVD in peritumoral liver tissue. High peritumoral co-expression of VEGF-C, VEGFR-1, and VEGFR-3 was associated with higher peritumoral macrophage density, determined by CD68 staining (0.87%±0.26% versus 0.45%±0.20%, *p* = 0.000, **upper**), and also had a much higher LVD (0.66%±0.20% versus 0.32%±0.25%, *p* = 0.012, **lower,** indicated as the D2-40-positive area); however, higher MVD was not observed (7.78%±0.40% versus 7.72%±0.45%, *p* = 0.870, **middle,** indicated as the CD31-positive area). Black bars, 50um.

Patients with high peritumoral co-expression of the three factors had a higher incidence of LNM (24/74 versus 13/108, *p* = 0.001). We also found a higher peritumoral lymphatic vessel density (LVD) in patients with a higher expression of the three factors (0.66%±0.20% versus 0.32%±0.25%, *p* = 0.012, as compared with the other patients, Figure 5); however, peritumoral MVD in the peritumoral tissues was not different in the patients with a higher expression of the three factors (7.78%±0.40% versus 7.72%±0.45%, *p* = 0.870, as compared with the other patients, **Figure 5** **).**

## Discussion

In the present study we demonstrated that high peritumoral co-expression of VEGF-C, VEGFR-1, and VEGFR-3 in patients with HCC was associated with a higher peritumoral distribution of macrophages, higher incidence of LNM, poorer overall survival and earlier tumor recurrence.

In contrast to many reports studying the role of angiogenesis- and lymphangiogenesis-related factors in tumor tissue [10], [11], [21], the present study explored the significance of the expression of these factors in peritumoral tissues. Although Yamaguchi et al. [22] suggested that the inflammatory status in the peritumoral area may enhance VEGF expression, our results did not support a relationship between the inflammatory status and the expression of the three factors in the peritumoral tissues. Furthermore, we found expression of VEGF-C, VEGFR-1, and VEGFR-3 in peritumoral tissues had a “centripetal distribution” pattern that was similar to the peritumoral distribution of M-CSF and macrophages observed in a previous study [4]. This distribution pattern of angiogenesis- and lymphangiogensis-related factors may be influenced by the tumor, probably by releasing cytokines or simply by compressing circulation and inducing hypoxia in surrounding liver tissues. It has been reported that interleukin-4 released from tumor cells attracts tumor-associated macrophages in the peritumoral tissue and induces cathepsin protease activity of macrophages to promote tumor growth and metastasis [23]. Hypoxia is also an important inducer of many cytokines in hepatocytes. One study showed hypoxic hepatocytes promote production of MMP-2 in stellate cells by releasing reactive oxygen species [24]. Another study showed hypoxia and proinflammatory factors upregulated apelin receptor in hepatocytes and led to an angiogenic response [25]. All the aforementioned cytokines in the peritumoral tissue may constitute a promotion for existing or developing metastatic tumor cells.

We found that patients with co-expression of VEGF-C, VEGFR-1, and VEGFR-3 in peritumoral tissue had short TTR, which may have been due to interactions between the peritumoral hepatocytes (VEGF-C/VEGFR-3 positive) and stromal cells (VEGFR-1 or VEGFR-3 positive). The VEGFR-1–expressing macrophages in the peritumoral stromal compartments could play an important role in the early recurrence by switching on regrowth of residual tumor cells after hepatectomy. As observed in other studies [26], macrophages can produce factors that promote tumor growth and metastasis, resulting in accelerated regrowth of residual tumor cells. Furthermore, the VEGF-C/VEGFR-3 axis of peritumoral hepatocytes leads to the autocrine loop and their own growth. The peritumoral hepatocyte clusters with abundant expression of VEGF-C could subsequently build a regulatory system to support the growth of macrophages via the VEGF-C/VEGFR-3 loop, similar to tumor growth promoted by the VEGF/VEGFR autocrine loop that is expressed on tumor cells [27]. Therefore, the VEGF-C/VEGFR-3 axis of peritumoral hepatocytes accompanied by macrophages expressing VEGFR-1 or VEGFR-3 could promote tumor recurrence.

In the present study, LNM, a significant prognostic factor, was also found to be associated with high peritumoral co-expression of VEGF-C, VEGFR-1, and VEGFR-3, which may be driven mostly by the peritumoral lymphangiogensis derived from interaction between the hepatocytes expressing VEGF-C/VEGFR-3 and stromal cells expressing VEGFR-3 or VEGFR-1. Several studies have shown that the macrophages in tumors, especially those in hypoxic and necrotic areas, are correlated with LNM and poor prognosis in other cancers [28], [29]. Skobe et al. [30]found VEGF-C from tumor cells could served as a chemoattractant for VEGFR-3–expressing macrophages, which in turn produced more VEGF-C and induced lymphangiogenesis via VEGFR-3 in lymphatic endothelial cells. In another scenario, lymphagiogenesis induced by macrophages was associated with angiogenesis in the induction of VEGF-A [14], which stimulated vascular endothelial cells via VEGFR-2, thus initiating angiogenesis and recruiting macrophages via its receptor VEGFR-1. However, in the present study, no statistical correlation was observed between peritumoral MVD and the populations with high peritumoral co-expression of the three factors; therefore, we considered that hepatocytes expressing VEGF-C may play a pivotal role in peritumoral lymphangiogenesis through interaction with the VEGFR-3 expression stromal cells such as macrophages and lymphatic endothelial cells. Furthermore, VEGFR-1–expressing macrophages may also provide chemotactic signals to attract VEGF-A–expressing tumor cells toward the gradient of VEGFR-1 in the peritumoral area, ultimately promoting migration and intravasation into the lymphatic vasculature and subsequent LNM [31]. Furthermore, the peritumoral lymphangiogensis still remained after hepatectomy and lymphadenectomy, which resulted in the early recurrence by regrowth of residual tumor cells in the liver or new LNM.

In summary, the peritumoral overexpression of VEGF-C, VEGFR-1, and VEGFR-3 may play an important role in HCC progression. Expression of the three factors in the peritumoral tissues may help to assess the risk of tumor recurrence in patients with HCC and optimize postoperative treatment to prevent tumor recurrence. Further studies should focus on the mechanisms of interaction between tumors and peritumoral tissues, which may help to develop targeted therapy for preventing postoperative early recurrence.

## Supporting Information

Table S1 **The median OS time and TTR among different combination of the five factors.**
(DOCX)

Table S2 **The median OS time and TTR for patients with different combinations of VEGF-C, VEGFR-1, VEGFR-3.**
(DOCX)
