# Supplementary material for: Prognostic Roles of Cross-Talk between Peritumoral Hepatocytes and Stromal Cells in Hepatocellular Carcinoma Involving Peritumoral VEGF-C, VEGFR-1 and VEGFR-3
Source: PLoS One. 2013 May 30;8(5):e64598. doi: 10.1371/journal.pone.0064598 (PMC3667811; doi:10.1371/journal.pone.0064598)
Supplement: Table S1 — The median OS time and TTR among different combination of the five factors. (DOCX) [file pone.0064598.s001.docx]

***Table S1.*** ***The median OS time and TTR among different combination of the five factors***

|  | **OS (months)** | | | **TTR (months)** | | |
| --- | --- | --- | --- | --- | --- | --- |
|  | **High** | **Not high** | ***p*** | **High** | **Not high** | ***p*** |
| **VEGF-A** | 21.8 | 41.9 | 0.310 | 16.9 | 31.3 | 0.512 |
| **VEGF-C** | 28.8 | 36.3 | 0.369 | 22.7 | 30.5 | 0.524 |
| **VEGFR-1** | 29.8 | 50.6 | 0.184 | 27.5 | 45.2 | 0.473 |
| **VEGFR-2** | 23.8 | 35.5 | 0.234 | 17.6 | 26.0 | 0.208 |
| **VEGFR-3** | 30.4 | 57.9 | 0.176 | 23.2 | 34.5 | 0.682 |
| **VEGF-A+VEGFR-1** | 25.6 | 36.8 | 0.550 | 20.5 | 26.6 | 0.775 |
| **VEGF-C+VEGFR-1** | 28.9 | 44.3 | 0.556 | 23.3 | 31.5 | 0.412 |
| **VEGFR-2+VEGFR-1** | 26.7 | 45.2 | 0.437 | 24.2 | 33.7 | 0.806 |
| **VEGFR-3+VEGFR-1** | 22.4 | 42.3 | 0.402 | 18.6 | 33.6 | 0.112 |
| **VEGF-A+VEGFR-3** | 26.4 | 43.7 | 0.451 | 22.7 | 30.3 | 0.420 |
| **VEGF-C+VEGFR-3** | 25.6 | 45.8 | 0.564 | 20.2 | 41.7 | 0.6503 |
| **VEGFR-2+VEGFR-3** | 26.8 | 44.0 | 0.308 | 23.3 | 33.5 | 0.714 |
| **VEGF-A+VEGFR-1+VEGFR-3** | 25.6 | 40.2 | 0.304 | 24.5 | 32.4 | 0.611 |
| **VEGF-C+VEGFR-1+VEGFR-3*** | 19.4 | 49.3 | 0.008 | 10.2 | 34.5 | 0.017 |
| **VEGFR-2+VEGFR-1+VEGFR-3** | 25.6 | 40.5 | 0.428 | 21.0 | 34.6 | 0.742 |
| **VEGF-A+VEGF-C+VEGFR-2+VEGFR-1** | 27.2 | 45.8 | 0.341 | 17.3 | 28.1 | 0.586 |
| **VEGF-A+VEGF-C+VEGFR-3+VEGFR-1** | 25.2 | 43.6 | 0.437 | 19.6 | 33.2 | 0.450 |
| **VEGF-C+VEGFR-2+VEGFR-3+VEGFR-1** | 22.6 | 44.8 | 0.550 | 16.2 | 34.2 | 0.483 |
| **VEGF-A+VEGFR-2+VEGFR-3+VEGFR-1** | 25.0 | 42.7 | 0.548 | 18.3 | 22.5 | 0.768 |
| **VEGF-A+VEGF-C+VEGFR-2+VEGFR-3** | 27.1 | 38.6 | 0.448 | 14.2 | 27.6 | 0.604 |
| **VEGF-A+VEGF-C+VEGFR-1+VEGFR-2+VEGFR-3** | 21.1 | 43.1 | 0.389 | 16.7 | 23.3 | 0.435 |
