# Supplementary material for: Prognostic Roles of Cross-Talk between Peritumoral Hepatocytes and Stromal Cells in Hepatocellular Carcinoma Involving Peritumoral VEGF-C, VEGFR-1 and VEGFR-3
Source: PLoS One. 2013 May 30;8(5):e64598. doi: 10.1371/journal.pone.0064598 (PMC3667811; doi:10.1371/journal.pone.0064598)
Supplement: Table S2 — The median OS time and TTR for patients with different combinations of VEGF-C, VEGFR-1, VEGFR-3. (DOCX) [file pone.0064598.s002.docx]

***Table S2.***

***The median OS time and TTR for patients with different combinations of VEGF-C,VEGFR-1,VEGFR-3***

|  | **OS (months)** | **TTR (months)** |
| --- | --- | --- |
| **VEGF-C^high^+VEGFR-1^high^+VEGFR-3^high^** | 19.4 | 10.2 |
| **VEGF-C^high^+VEGFR-1^low^+VEGFR-3^low^** | 49.2 | 34.6 |
| **VEGF-C^low^+VEGFR-1^high^+VEGFR-3^low^** | 48.4 | 35.0 |
| **VEGF-C^low^+VEGFR-1^low^+VEGFR-3^high^** | 45.3 | 36.1 |
| **VEGF-C^high^+VEGFR-1^high^+VEGFR-3^low^** | 44.2 | 34.2 |
| **VEGF-C^high^+VEGFR-1^low^+VEGFR-3^high^** | 47.9 | 36.9 |
| **VEGF-C^low^+VEGFR-1^high^+VEGFR-3^high^** | 46.2 | 35.2 |
| **VEGF-C^low^+VEGFR-1^low^+VEGFR-3^low^** | 45.1 | 34.4 |
